# Multiparametric dynamic whole-body PSMA PET/CT using [^68^Ga]Ga-PSMA-11 and [^18^F]PSMA-1007

**DOI:** 10.1186/s13550-023-00981-8

**Published:** 2023-04-15

**Authors:** André H. Dias, Mads R. Jochumsen, Helle D. Zacho, Ole L. Munk, Lars C. Gormsen

**Affiliations:** 1 Department of Nuclear Medicine & PET Centre, Aarhus University Hospital, Palle Juul-Jensens Boulevard 165, 8200 Aarhus N, Denmark; 2 Department of Nuclear Medicine and Clinical Cancer Research Centre, Aalborg University Hospital, Aalborg, Denmark; 3 Department of Clinical Medicine, Aarhus University, Aarhus, Denmark; 4 Department of Clinical Medicine, Aalborg University, Aalborg, Denmark

**Keywords:** Dynamic whole-body PET, PSMA, Parametric imaging, Patlak, Oncology, Prostate cancer

## Abstract

**Background:**

Routine prostate-specific membrane antigen (PSMA) positron emission tomography (PET) performed for primary staging or restaging of prostate cancer patients is usually done as a single static image acquisition 60 min after tracer administration. In this study, we employ dynamic whole-body (D-WB) PET imaging to compare the pharmacokinetics of [^68^Ga]Ga-PSMA-11 and [^18^F]PSMA-1007 in various tissues and lesions, and to assess whether Patlak parametric images are quantitative and improve lesion detection and image readability.

**Methods:**

Twenty male patients with prostate cancer were examined using a D-WB PSMA PET protocol. Ten patients were scanned with [^68^Ga]Ga-PSMA-11 and ten with [^18^F]PSMA-1007. Kinetic analyses were made using time-activity curves (TACs) extracted from organs (liver, spleen, bone, and muscle) and lesions. For each patient, three images were produced: SUV + Patlak parametric images (*K*_*i*_ and DV). All images were reviewed visually to compare lesion detection, image readability was quantified using target-to-background ratios (TBR), and *Ki* and DV values were compared.

**Results:**

The two PSMA tracers exhibited markedly different pharmacokinetics in organs: *reversible* for [^68^Ga]Ga-PSMA-11 and *irreversible* for [^18^F]PSMA-1007. For both tracers, lesions kinetics were best described by an irreversible model. All parametric images were of good visual quality using both radiotracers. In general, *Ki* images were characterized by reduced vascular signal and increased lesion TBR compared with SUV images. No additional malignant lesions were identified on the parametric images.

**Conclusion:**

D-WB PET/CT is feasible for both PSMA tracers allowing for direct reconstruction of parametric *Ki* images. The use of multiparametric PSMA images increased TBR but did not lead to the detection of more lesions. For quantitative whole-body *Ki* imaging, [^18^F]PSMA-1007 should be preferred over [^68^Ga]Ga-PSMA-11 due to its irreversible kinetics in organs and lesions.

**Supplementary Information:**

The online version contains supplementary material available at 10.1186/s13550-023-00981-8.

## Introduction

Prostate cancer (PCa) is one of the most frequently occurring cancers in the male population [1] and is now the second most common cause of death from malignancy in this group [2]. Positron emission tomography (PET) is an integral part of PCa management, with prostate-specific membrane antigen (PSMA)-targeted tracers used for primary staging and also in the evaluation of biochemical relapse, assisting in therapy planning and disease management [3, 4].

PSMA is a cellular transmembrane surface protein that is overexpressed in PCa. Unfortunately, PSMA is not prostate-specific [5] and also binds to cells in other tissues as well as to the neovasculature in other malignancies [6]. Several PSMA radiotracers have been produced [7]. Labelled predominantly with ^68^Galium (^68^Ga) and ^18^Fluoride (^18^F), these radiotracers have been made commercially available and disseminated in clinical trials and daily clinical practice. The use of ^68^Ga radiolabelled compounds is widely implemented. However, production of ^68^Ga-radiotracers requires an on-site ^68^Ga generator, and ^68^Ga radiotracers have a relatively shorter half-life and longer positron range than ^18^F labelled tracers, resulting in images with lower spatial resolution.

Conventional static PSMA PET imaging is usually performed 60 min after tracer administration, although modified protocols with scanning at different time points have been suggested to improve image quality [8, 9]. However, most previous studies have been limited by the single timepoint evaluation of conventional PET, and only few comprehensive studies of PSMA pharmacokinetics have been attempted. These have mostly applied a dynamic protocol on conventional field of view cameras constrained to two bed positions over the pelvis [10–15], with only one truly “whole-body” dynamic acquisition study performed on a total-body PET/CT scanner [16]. Using dynamic PET with [^68^Ga]Ga-PSMA-11 has shown to increase identification rates of both primary PCa and local recurrence [12, 17], suggesting that dynamic whole-body (D-WB) PSMA PET imaging could outperform static PSMA PET.

For the analysis of PSMA kinetics, a two-tissue compartment model can be applied to describe the tracer exchanges between plasma and tissue. In this case, the first compartment (unbound compartment) represents the free unbound tracer in interstitial fluid, while the second compartment (bound compartment) represents tracer bound to the PSMA receptors, with the transport rate k_3_ correlating with tracer binding and internalization, and k_4_ with the dissociation of the tracer from the receptor and externalization. For PCa lesions, the k4 ≈ 0, as the binding is predominantly irreversible [11].

Previously, dynamic PET acquisition was limited to a single field of view and technically difficult to perform due to the necessity of acquiring arterial blood samples to accurately determine the input function. Given the disseminated nature of malignancies such as PCa and the volume of patients referred for pre-therapy PET, dynamic PET has not been implemented in routine oncological work-up. However, this may be about to change.

Recently introduced methodology [18, 19] allows for D-WB PET acquisitions in conventional PET scanners, by applying the linear Patlak model [20, 21] to a multi-pass continuous WB dynamic PET acquisition. This imaging protocol provides not only the conventional standardized uptake value (SUV) images, but also multiparametric images based on Patlak kinetic modelling [22]. These images are the *Ki* images (representing the effective tracer binding by the PSMA receptors) and DV images (representing the distribution volume of non-trapped tracer in the reversible compartments and the fractional blood volume). These advances have prompted renewed interest and research in the use of dynamic PET for oncological imaging as reviewed elsewhere [23, 24]. While the multiparametric D-WB protocol has been successfully applied for 2-[^18^F]fluoro-2-deoxy-D-glucose ([^18^F]FDG) [25–27], it has until now only to a lesser degree been explored for other tracers.

At our department, we recently transitioned from a ^68^Ga labelled tracer ([^68^Ga]Ga-PSMA-11) to an ^18^F labelled tracer ([^18^F]PSMA-1007) for routine clinical PSMA PET allowing for a comparison of the two radiotracers. With this study, we therefore aimed to evaluate the tissue pharmacokinetics of these two tracers, the image quality and clinical impact of multiparametric D-WB PSMA PET imaging, and the quantitative accuracy of the resulting parametric values.

## Materials and methods

### Patient population

This study was a retrospective analysis of data. Participants were recruited from all patients referred for PSMA PET/CT as part of their clinical evaluation if they were deemed fit to lie still for 70 min during scanning. The study was approved by the local ethics committee in the Central Denmark Region (1-10-72-188-19).

D-WB PSMA data were obtained from 20 male patients with known prostate cancer. Ten patients were scanned with [^68^Ga]Ga-PSMA-11 and ten patients with [^18^F]PSMA-1007.

### Data acquisition and image reconstruction

The study participants were scanned on a Siemens Biograph Vision 600 PET/CT scanner (Siemens Healthineers, Knoxville, USA) with a 26.2-cm axial field of view. A fully automated multiparametric PET/CT acquisition protocol (Multiparametric PET Suite AI, Siemens Healthineers, Knoxville, USA) was used.

*[*^*68*^*Ga]Ga-PSMA-11 cohort (N* = *10)*: These subjects were scanned with a 76-min multiparametric PET acquisition protocol, started at the time of an injection of [^68^Ga]Ga-PSMA-11 (2 MBq/kg). The PET protocol consisted of 1) a 6-min dynamic scan with the bed fixed at the chest region, and 2) a 70-min dynamic WB PET scan consisting of seven continuous 10-min WB passes.

*[*^*18*^*F]PSMA-1007 cohort (N* = *10)*: A 70-min multiparametric PET acquisition protocol was started at the time of an injection of [^18^F]PSMA-1007 (2 MBq/kg). The PET protocol consisted of 1) a 6-min dynamic scan with the bed fixed at the chest region, and 2) a 64-min dynamic WB PET scan consisting of 16 continuous bed motion passes: 7 × 2-min WB passes followed by 9 × 5-min WB passes.

The dynamic image acquisition protocols were therefore not entirely identical. In practice, this meant that 40–70 min direct parametric reconstructions for [^68^Ga]Ga-PSMA-11 were performed on 3 10-min images, while for [^18^F]PSMA-1007 they were calculated from 6 5-min images. However, since all multiparametric images were based on 30-min D-WB PET data, we expect this variation of the frame length to have a minimal impact on image quality and noise of the multiparametric images (*Ki* and DV) [18].

For both tracers, multiparametric images (*Ki* and DV) were reconstructed using the data from 40-to-70-min post-injection and the image-derived input function (IDIF). This reconstruction protocol was performed using the direct Patlak reconstruction in the Multiparametric PET Suite AI software from Siemens Healthineers. A standard-of-care static SUV image was reconstructed using data from 60-to-70-min post injection. The PET reconstruction parameters for D-WB: For the 10-min SUV image, we used TrueX + time-of-flight, four iterations, five subsets, 440 × 440 matrix, 2-mm Gaussian filter, and relative scatter correction (reconstruction time 2.5 min). Parametric images of *Ki* and DV were generated using the direct Patlak reconstruction method with non-negativity constraints using list-mode data from multiple passes (40–70 min), TrueX + time-of-flight, eight iterations, five subsets, 30 nested loops, 440 × 440 matrix, 2-mm Gaussian filter, and relative scatter correction (reconstruction time 13.5 min). For image-based kinetic analyses, we also made a 0–6-min dynamic series of the chest region (12 × 5 s, 6 × 10 s, 8 × 30 s; reconstruction time 5 min), and a 6–70-min dynamic WB series (16 passes, reconstruction time 23 min), using the same reconstruction parameters as the static SUV image. This results in complete 0–70-min dynamic PET data coverage of the chest region.

After image acquisition, the automated multiparametric scan protocol automatically identified the aorta on the low-dose WB CT scan a technology from Siemens Healthineers known as automated learning and parsing of human anatomy (ALPHA) [28] and placed a cylindric volume of interest (VOI) (1.6 mm^3^) on the descending aorta to extract the IDIF from the full dynamic PET series of the chest region. Such an IDIF is robust and can be used to replace an arterial blood input function for precise quantitative Patlak modelling [29].

### Image analysis and VOI delineation

Multiparametric images were visually evaluated by two nuclear medicine physicians using Hermes Gold Client v.2.5.0 (Hermes Medical Solutions AB, Stockholm, Sweden). VOI delineation of the multiparametric images was performed using PMOD® 4.0 (PMOD Technologies Ltd, Zürich, Switzerland). Semiquantitative values of SUV_max_ and SUV_mean_ were obtained from the conventional PET reconstructions, whereas *Ki* and DV values were extracted from the multiparametric images.

For each patient, VOIs were analysed from areas of tissue without evidence of pathology. Specifically, we performed delineation of an area of the liver, spleen, parotid gland, lacrimal gland, healthy bone, muscle, benign ganglia (with active PSMA signal) in the pelvis and thorax, and bladder. Areas with pathologically increased uptake of PSMA were identified and delineated using an isocontouring method of 55% of SUV_max_ in the VOI [30]. Thus, we outlined the primary tumour in the prostate, as well as lymph node and bone lesions. In patients with an uncountable number of active lesions, for example in disseminated skeletal disease, up to ten individual foci were chosen for delineation. Background regions were delineated in the vicinity of these target lesions, corresponding to an elongated ROI drawn in adjoining tissue in at least three consecutive slices. The individual methodology used to delineate these areas can be found in Additional file 1: Table S1, and an example of a lesion and background delineation can be found in Additional file 1: Fig. S1.

We used target-to-background ratio (TBR) as an objective metric for quantitative assessment of ‘lesion detectability’. Detectable lesions require a TBR > 1, and a higher TBR indicates better lesion detectability.

### Comparison of multiparametric and image-based Ki and DV values

The estimates of kinetic parameters obtained through indirect image-based analysis can differ from those obtained by direct reconstruction of parametric images, with the latter exhibiting more favourable bias and noise characteristics, as demonstrated in reference [31]. The noise and bias in *Ki* images are influenced by factors such as the specific implementation of the optimization algorithm, the mathematical formulation of the Patlak model, and the utilization of non-negativity constraints [32, 33]. We therefore compared the kinetic parametric estimates using the two methods. The image-based parameters were calculated by linear Patlak analysis in PMOD® 4.0, using the general kinetic modelling tool (PKIN) with the lumped constant set to 1 and discarding fits with negative values. The direct reconstructed values were obtained using the Multiparametric PET Suite AI from Siemens Healthineers.

### Kinetic analysis

70-min dynamic scan data from the fixed bed at the chest region was analysed using a two-tissue compartment model (2CM) and the 70-min IDIF using PMODs PKIN module. More specifically we analysed VOIs in the liver, spleen, healthy bone (thoracal vertebra), muscle (paravertebral) and any of the previously delineated PSMA avid lesions that were included in this limited scan field-of-view. Parameter estimates for a reversible (*k*_4_ > 0) and irreversible (*k*_4_ = 0) 2CM were obtained and compared with the parameters from the irreversible Patlak model [20] and the reversible Logan Model [34]. Akaike information criterion (AIC) [35] was used to select the CM that best fitted each tissue and for each tracer.

### Statistical analysis

Statistical analyses were performed using GraphPad Prism 9.2.0. Statistical tests were used for group comparisons (paired/unpaired) and to assess whether data were normally distributed. Welch’s T test was performed for normal distributed data (liver, spleen, bone, and benign ganglia), while the Mann–Whitney test was performed for non-normal distributed data (prostate lesions, lymph node lesions, bone lesions, parotid gland, lacrimal gland, and muscle).

Pearson’s correlation analysis was performed for the relation between *Ki* and SUV values. *P* values of < 0.05 were considered significant. Continuous group data are presented as mean ± SD or median (range) as appropriate. Time-series are presented as mean ± SEM.

## Results

### Time-activity curves

**Fig. 1:**
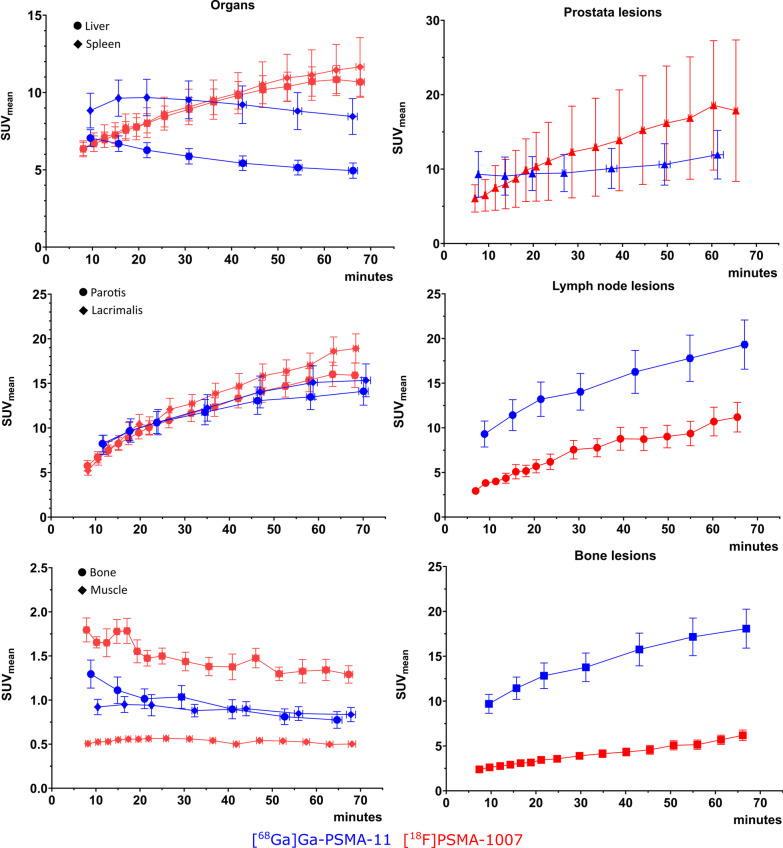
Representation of time activity curves for SUV_mean_ values. In blue are the TACs for [^68^Ga]Ga-PSMA-11, in red the curves for [^18^F]PSMA-1007. On the left column, the plots for healthy organs: Above: liver and spleen; middle: parotid gland and lacrimal gland; below: bone and muscle. On the right column: Above: prostate lesions; middle: lymph node lesions; below: skeletal lesions

The whole-body dynamic series contained TACs of the analysed organs as well of the pathological findings as presented in Fig. 1. Lesions displayed continuously increasing PSMA activity over time, independent of the tracer used. However, there was a difference in tracer behaviour when analysing the healthy organ areas. While gradual increase (for parotid or lachrymal glands) or decrease (for bone and muscle) was observed for both tracers, the kinetics of hepato-splenic activity appeared to differ between tracers. Where [^68^Ga]Ga-PSMA-11 showed decreasing activity in the liver and spleen, a gradually increasing PSMA activity over time was seen with [^18^F]PSMA-1007.

### Full kinetic analysis using 2CM

The two PSMA tracers exhibited significantly different pharmacokinetics in normal organs (liver, spleen, bone, and muscle) as measured by AIC values. For [^68^Ga]Ga-PSMA-11, the normal organ TACs were best fitted using the reversible 2CM. For [^18^F]PSMA-1007, the normal organ TACs were best fitted using the irreversible 2CM except for muscle that were best fitted using a reversible model. For both tracers, the lesions were best fitted using the irreversible 2CM. The AIC values are shown in Additional file 1: Fig. S2.

**Fig. 2:**
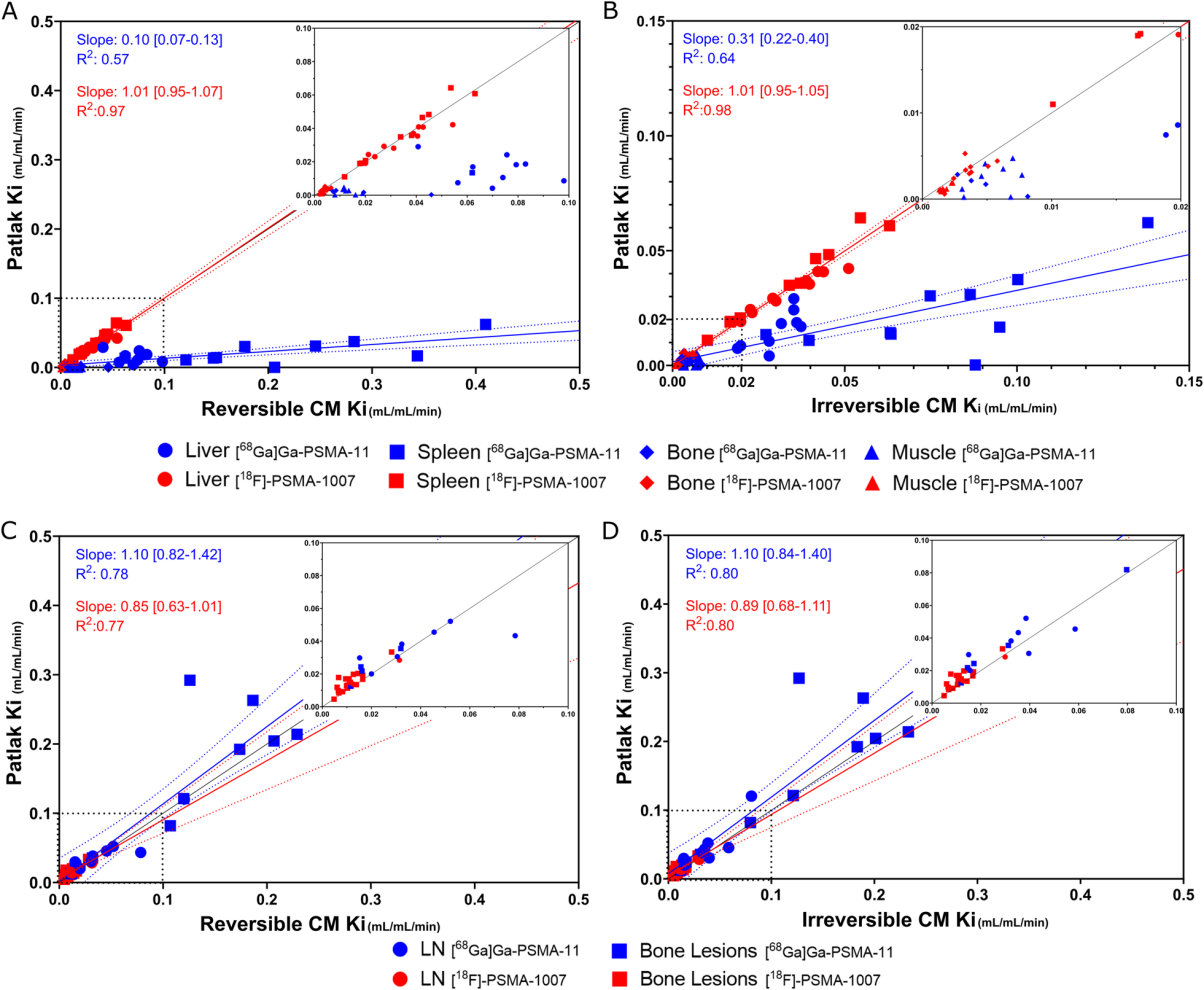
Correlation between values from the parametric Ki images as function of the Ki values from full kinetic analyses using the reversible 2CM (left) and irreversible 2CM (right). In blue are the data for [^68^Ga]Ga-PSMA-11, in red [^18^F]PSMA-1007. For the analysed organs (**A** and **B**), excellent correlation is seen for [^18^F]PSMA-1007, whereas for [^68^Ga]Ga-PSMA-11, the correlation was poor, and the multiparametric Ki values were strongly biased. For lymph node and bone lesions (**C** and **D**), excellent correlation is seen for both tracers

The multiparametric images are based on the Patlak model that assumes irreversible kinetics. For normal organs, Fig. 2 shows the correlation between values from the parametric *Ki* images as function of the *Ki* values from full kinetic analyses using the reversible and irreversible 2CM, respectively. For [^18^F]PSMA-1007, there was excellent correlations, whereas for [^68^Ga]Ga-PSMA-11, the correlation was poor, and the multiparametric *Ki* values were strongly biased. However, for [^68^Ga]Ga-PSMA-11, we found good correlation between the total distribution volume calculated using Logan analysis and full kinetic modelling using the reversible 2CM (Additional file 1: Fig. S3A, B) as expected for organs with reversible kinetics. For lesions, the correlations between the *Ki* estimates were excellent for both tracers as seen in Fig. 2 and Additional file 1: Fig. S3C.

### Comparison of multiparametric and image-based Ki values

For both [^68^Ga]Ga-PSMA-11 and [^18^F]PSMA-1007, we found excellent correlation (*r*^2^ = 0.99, *p* < 0.0001) between *Ki* values extracted from the multiparametric images (direct reconstruction) versus image-based calculations performed in PMOD’s PKIN module (Additional file 1: Fig. S4).

### Visual analysis of images

The clinical indications and characteristics of the study subjects are shown in Table 1.

**Table 1:** Characteristics of the study population

Patient no	Age	Indication	Previous treatment or other notes	PSA (µg/L)	Gleason	Tracer	Dose (MBq)
1	70	Primary staging		6.4	7	[^68^Ga]Ga-PSMA-11	151
2	53	Primary staging		7.2	7	[^68^Ga]Ga-PSMA-11	151
3	68	Primary staging		30	7	[^68^Ga]Ga-PSMA-11	163
4	67	Relapse	Prostatectomy and LN extraction	4	8	[^68^Ga]Ga-PSMA-11	198
5	78	Primary staging		11	7	[^68^Ga]Ga-PSMA-11	182
6	63	Primary staging	Increased prostate size, negative previous biopsies	35	NA	[^68^Ga]Ga-PSMA-11	135
7	60	Primary staging		47	8	[^68^Ga]Ga-PSMA-11	177
8	71	Primary staging		95	9	[^68^Ga]Ga-PSMA-11	175
9	57	Primary staging		160	7	[^68^Ga]Ga-PSMA-11	204
10	69	Primary staging		14.6	7	[^68^Ga]Ga-PSMA-11	194
11	75	Progression	Medical castration	707.7	7	[^18^F]PSMA-1007	151
12	73	Relapse	Prostatectomy	0.2	8	[^18^F]PSMA-1007	201
13	66	Progression	Active surveillance	9.6	6	[^18^F]PSMA-1007	181
14	63	Primary staging		32.5	8	[^18^F]PSMA-1007	209
15	64	Relapse	Prostatectomy + RT	0.2	7	[^18^F]PSMA-1007	197
16	71	Primary staging	Increased prostate size, negative previous biopsies	14	NA	[^18^F]PSMA-1007	240
17	65	Primary staging		72.4	8	[^18^F]PSMA-1007	236
18	78	Primary staging		31.6	7	[^18^F]PSMA-1007	205
19	68	Primary staging		5.1	9	[^18^F]PSMA-1007	205
20	71	Primary staging		5.9	7	[^18^F]PSMA-1007	164

**Fig. 3:**
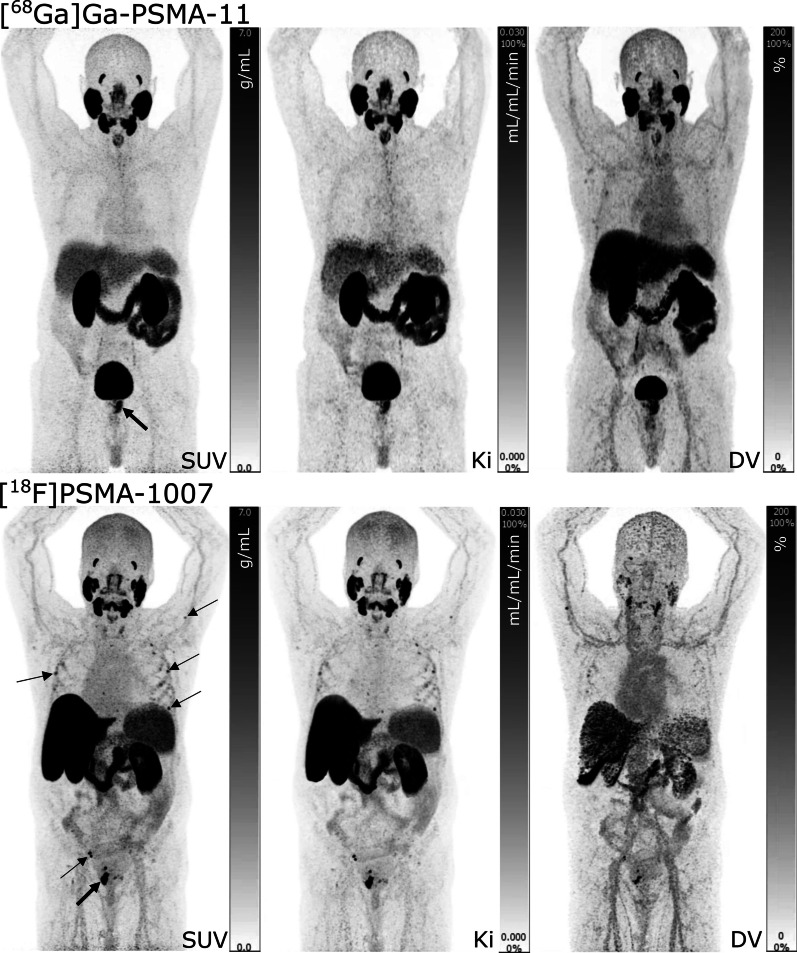
Examples of D-WB PET/CT scans performed in patients referred for primary staging of prostate cancer. SUV images are reconstructed using D-WB data from 60 to 70 min, whereas the parametric images of Ki and DV are reconstructed using D-WB data from 40 to 70 min. Above: the example of a D-WB PET/CT scan performed with [^68^Ga]Ga-PSMA-11 displaying only primary disease (arrow). Below: one of the patients scanned with [^18^F]PSMA-1007 showing primary disease in the prostate (thick arrow), dissemination to pelvic lymph nodes, as well as multiple small skeletal lesions in the ribs and left humerus (probably unspecific/benign) (thin arrows)

All D-WB PET images were of good visual quality as shown in the examples below (Fig. 3).

In general, PSMA *Ki* images were characterized by reduced signal in vascularized tissues (liver, mediastinum, spleen, and large vessels). The DV images were characterized by high signal intensity in the vascularized organs and also by the presence of signal from both benign and malignant lesions. The vascular [^18^F]PSMA-1007 signal displayed on DV images appears to be more intense than that of [^68^Ga]Ga-PSMA-11.

The *[*^*68*^*Ga]Ga-PSMA-11 cohort* consisted of nine primary staging and one relapse evaluations. Malignant disease was found in 9/10 scans. One primary staging patient had localized prostatic disease, while seven patients had disease dissemination to lymph nodes and/or bone. The relapse study patient showed disseminated lymph node disease. A total of 274 VOIs were delineated (including healthy organs, targets, benign ganglia, and background areas).

The *[*^*18*^*F]PSMA-1007 cohort* consisted of six primary staging evaluations, two disease progression studies, and two relapse evaluations. Malignant disease was found in 9/10 scans. In the staging and progression scans, one patient presented with localized prostatic disease, and the rest with disseminated disease. One patient with suspected disease relapse showed no signs of active disease, whereas the other had bone metastases. A total of 210 VOIs were delineated.

In all 20 patients, visual lesion detection in terms of the number of identified lesions was almost identical between SUV and parametric images, with a few notable exceptions. In two patients, SUV images revealed PSMA avid foci in soft tissue of the shoulder region, as shown in Additional file 1: Fig. S5. These foci were absent from the *Ki* images and visible on the DV images indicating (correctly) that the SUV image PSMA avidity was due to free tracer.

One patient was diagnosed with a hepatocelular carcinoma of the liver. As seen in Additional file 1: Fig. S6, although the *Ki* images reduced liver background activity and thus improved TBR, the liver lesion was in fact more easily identified on SUV and DV images.

### Quantitative analysis of lesion detectability

**Fig. 4:**
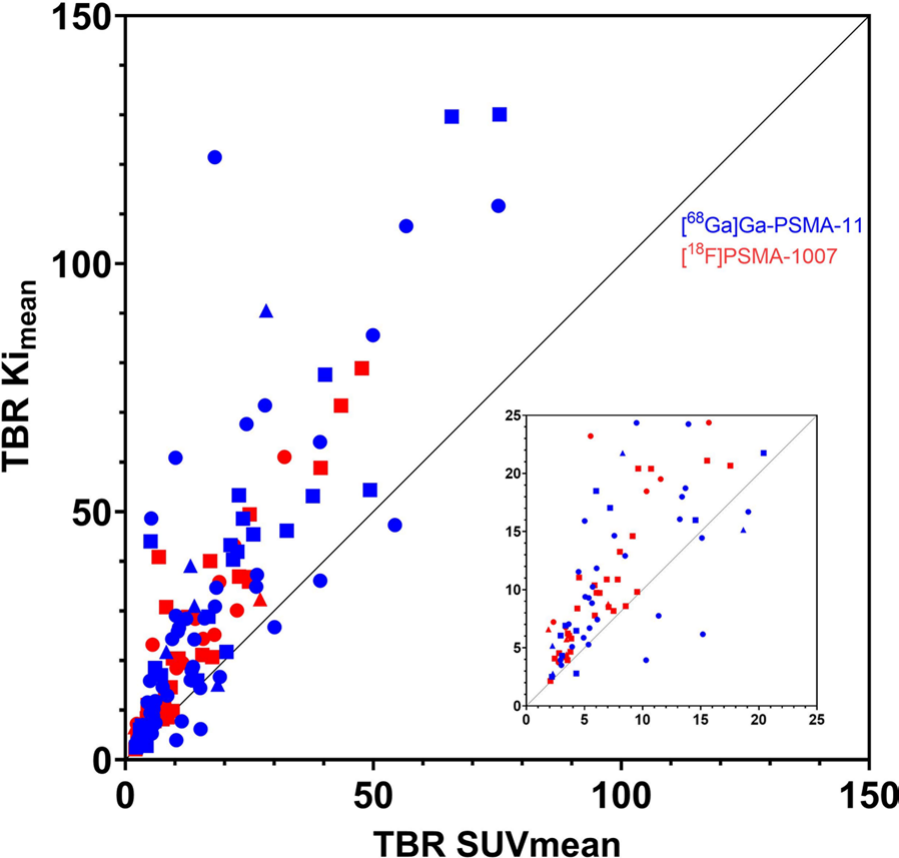
Distribution of analysed VOIs showing a clear predominance of lesions favouring the tumour-to-background ratio in the parametric images. Main plot with distribution of all VOIs, insert with zoom on area 25 × 25 TBR. The triangle (△) symbol represents prostate lesions, circle (○) symbol represents lymph node lesions, square (□) symbol represents bone lesions

As expected, [^18^F]PSMA-1007 prostate and bladder TBR was better than [^68^Ga]Ga-PSMA-11 (Additional file 1: Fig. S7). In addition, the observed lesion TBR was superior in the *Ki* images as indicated by the location of 135/145 lesions above the line of identity (Fig. 4). In ten lesions, TBR SUV_mean_ > TBR *Ki*_mean_, all of which were [^68^Ga]Ga-PSMA-11 PET/CT scans (eight lymph nodes, one bone lesion, one prostate lesion).

**Fig. 5:**
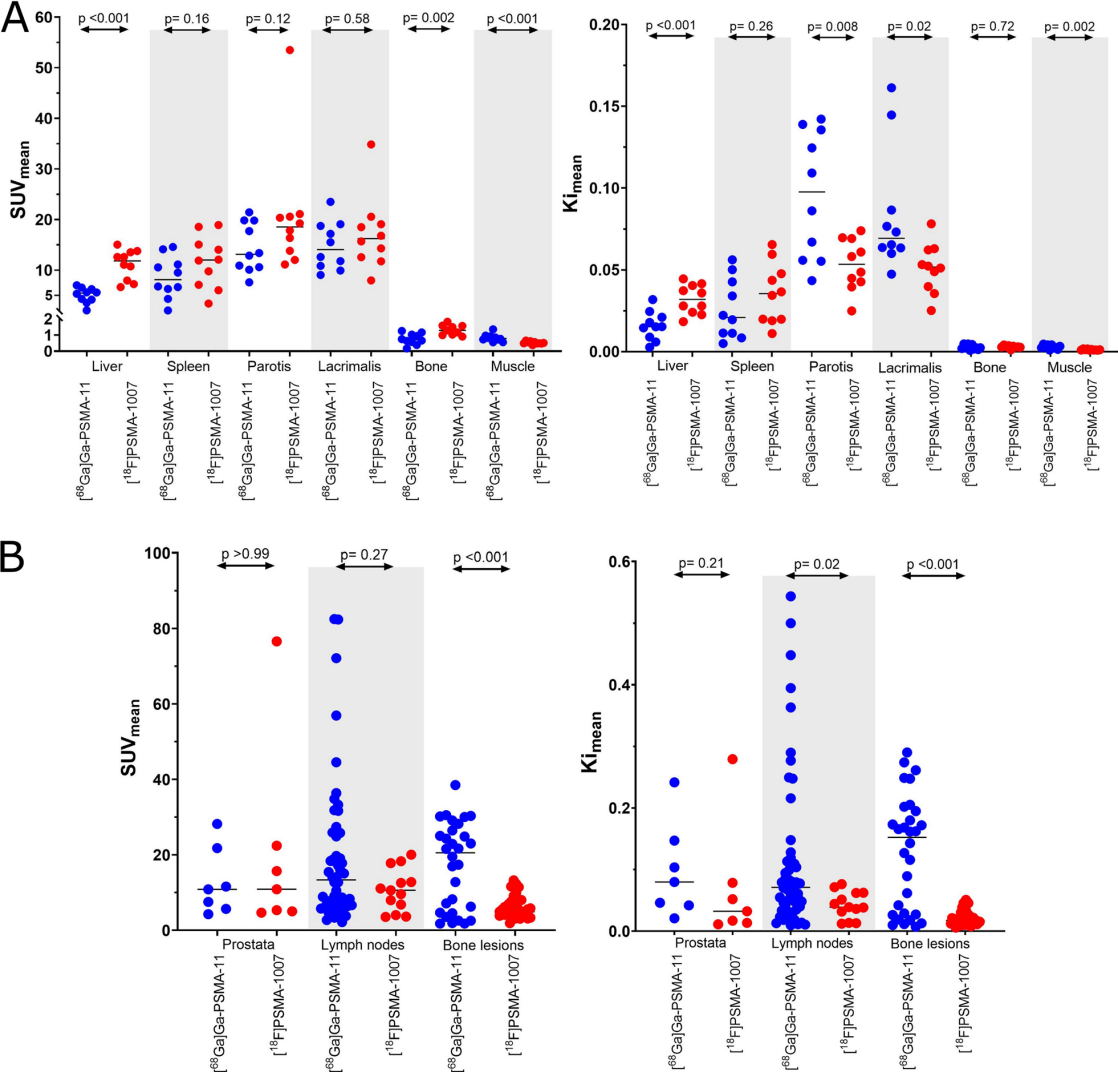
Distribution of analysed volumes of interest **A** in healthy organs; **B** for malignant lesions in the prostate, lymph nodes and skeletal structures. Represented are SUV_mean_ (on the left) and Ki_mean_ (on the right) for both tracers. [^68^Ga]Ga-PSMA-11 in represented in blue, [^18^F]PSMA-1007 in red

The SUV_mean_ in the liver, bone and muscle was significantly different (*p* < 0.05) between [^68^Ga]Ga-PSMA-11 and [^18^F]PSMA-1007. For the parametric images, *Ki*_mean_ values differed significantly between the two tracers in the liver, muscle, parotid and lacrimal glands. We saw no significant difference in the distribution of SUV_mean_ activity between tracers in the primary tumour of the prostate, in lymph nodes or in the physiologic uptake in ganglia. However, the skeletal lesions showed significantly lower activity with [^18^F]-PSMA-1007. *Ki*_mean_ values were significantly greater using [^68^Ga]Ga-PSMA-11 than [^18^F]PSMA-1007 for nodal and bone lesions but not in the prostate or in benign ganglia (Fig. 5; data for benign ganglia is show in Additional file 1: Fig. S8, data for SUV_max_ is shown in Additional file 1: Fig. S9).

**Fig. 6:**
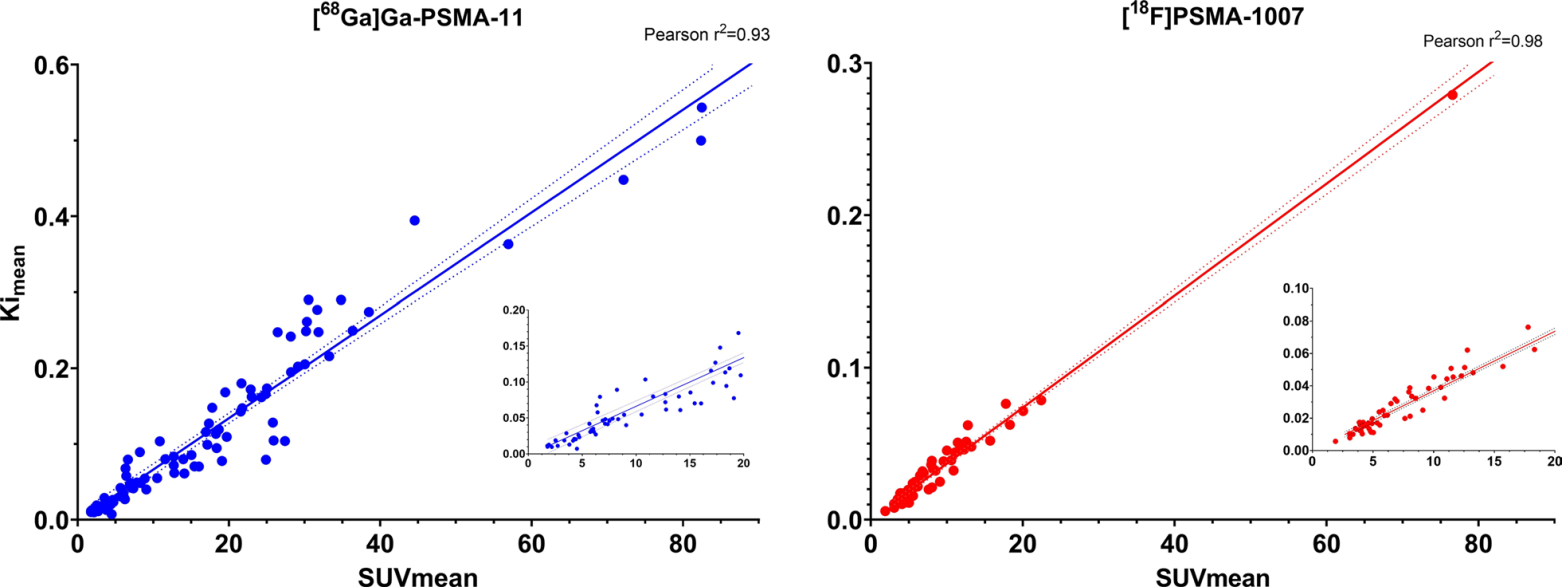
Correlation of SUV_mean_ and Ki_mean_ for lesions. On the left: [^68^Ga]Ga-PSMA-11; on the right: [^18^F]PSMA-1007. Inserts containing the lower ranges of SUV_mean_ and Ki_mean_ can be seen on both plots

An excellent correlation between quantitative (*Ki*_mean_) and semi-quantitative (SUV_mean_) measurements of lesion metabolism were seen for both tracers (Fig. 6), slightly higher for [^18^F]PSMA-1007 ([^68^Ga]Ga-PSMA-11: *r*^2^ = 0.93; [^18^F]PSMA-1007: *r*^2^ = 0.98).

## Discussion

PSMA multiparametric PET images for both radiotracers were of good visual quality as reflected by the excellent TBR and overall image appearance. In general, lesion TACs were roughly similar between the two radiotracers, whereas organ TACs differed noticeably due to the different modes of excretion. Finally, the parametric values derived from the image-based kinetic analyses compared well with the parametric images using direct reconstruction allowing for simple acquisition of whole body PSMA kinetics.

Multiparametric *Ki* imaging assumes an irreversible kinetic model in organs and lesions. Tissues with reversible uptake will have underestimated *Ki* depending on the degree of reversibility that may differ between tissues. We found that the multiparametric *Ki* values for lesions strongly correlated with *Ki* values obtained from full 2CM analysis for both tracers, whereas the multiparametric *Ki* values for normal organs, such as liver, spleen, and bone, were quantitative only for [^18^F]PSMA-1007. Overall, these results indicate that [^18^F]PSMA-1007 is better suited for quantitative multiparametric *Ki* imaging than [^68^Ga]Ga-PSMA-11 as more organs exhibit irreversible kinetics. The observed difference can perhaps be attributed to the different binding potentials of the two tracers [36, 37]. Muscle tissue kinetics were best analysed by reversible 2CM for both tracers, probably due to the low muscular PSMA activity [5]. For [^18^F]PSMA-1007, *Ki* values were quantitative both in healthy bone and bone lesions, which could allow for quantification of disease progression. This may be of clinical interest since the threshold for pathology on static SUV images has been hard to establish due to the varying “normal” PSMA uptake in bones. Thus, we recommend using [^18^F]PSMA-1007 for quantitative multiparametric *Ki* imaging in order to obtain images with unbiased quantifications of background organs and tissues, whereas both tracers can be used for lesion detection.

The main advantage of PSMA D-WB multiparametric images over static SUV images is that the former allow for differentiation between free unbound tracer (background) and tracer bound to the PSMA receptors. *Ki* images are therefore characterized by improved target-to-background ratio, which in theory could improve lesion identification. However, even though tumour-to-background ratios were in general more favourable on our *Ki* images, we identified no additional pathologic lesions in this patient cohort. This result is similar to that of previous larger studies [11, 14] and attests to the known robustness of conventional SUV imaging.

[^18^F]PSMA-1007 is often preferred by clinical departments due to its supposedly superior properties evaluating pathology in the pelvis, and the absence of reliance on an onsite gallium-68 generator. However, in our hands, both PSMA tracers identified primary prostatic disease with ease, regardless of whether images were static SUV or parametric *Ki* images. However, the improvement in regional TBR contrast associated with [^18^F]PSMA-1007 is likely to be more clinically relevant in relapse evaluation studies, of which we only had three in our cohort. Furthermore, it is recommended to administer furosemide shortly before or after administration of [^68^Ga]Ga-PSMA-11 [38], thus diminishing the high residual activity in the bladder [39]. Our protocol lacks such intervention, as it would reduce patient compliance with the prolonged scan time. Finally, this disadvantage associated with [^68^Ga]Ga-PSMA-11 scanning can also be circumvented using D-WB imaging which provides better TBR (in this case PCa to bladder) of the *early* dynamic images [12, 15, 40]. Whether this potentially translates to improved detection of recurrent disease by D-WB [^68^Ga]Ga-PSMA-11 remains to be clarified in a larger study.

Both radiotracers readily identified lymph node metastases, although with higher *Ki* values in the [^68^Ga]Ga-PSMA-11 images. Likewise, skeletal lesion [^68^Ga]Ga-PSMA-11 activity was greater in static SUV images and calculated *Ki* values were higher in the parametric images. Coupled with the generally higher background signal in the bone observed in [^18^F]PSMA-1007 PET images, these findings seem to suggest that [^68^Ga]Ga-PSMA-11 PET should outperform [^18^F]PSMA-1007 PET in both lymph node and bone lesion detection. However, previous head-to-head studies based on static images have shown more bone lesions detected using static [^18^F]PSMA-1007 PET than [^68^Ga]Ga-PSMA-11. This is now a known disadvantage of [^18^F]PSMA-1007, as up to half of these additional bone ‘lesions’ have turned out to be benign [41, 42], and consequently, no difference in radiotracer sensitivity to detect malignant skeletal lesions have been reported [43, 44]. Although histological verification was not available in all lesions in the current study, it is evident to us that some of the delineated bone lesions with [^18^F]PSMA-1007, particularly those in the ribs and with low SUV values, likely also represent unspecific benign lesions [41, 42]. The presence of such unspecific bone lesions can contribute to the difference in distribution of SUV signal observed in Fig. 5, as the [^68^Ga]Ga-PSMA-11 cohort included a larger amount of likely bone metastases. The bone lesions were also visible on parametric PSMA PET using both radiotracers, which is unsurprising even though these lesions are probably visualized due to a non-PSMA-related uptake mechanism [45].

Consequently, parametric images cannot be used to differentiate these unspecific bone lesions from malignant disease, regardless of radiotracer used or scan protocol employed.

Whereas sensitivity to detect lesions was not improved by the parametric imaging, specificity appears to be slightly better. In our cohort of 20 patients, we observed two cases of ‘false-positive’ findings in soft-tissue lesions on the SUV images that showed no tracer uptake on the parametric reconstructions, as previously reported for [^18^F]FDG [25]. However, it is relevant to note that the presence of isolated soft-tissue or lymph node metastasis in the upper extremities is highly unlikely in prostate cancer.

Some limitations to the study must be acknowledged. First, the patient cohort is rather small, and for ethical reasons, patients were not subjected to repeat studies using the two different radiotracers. A direct comparison of findings in each study was therefore not possible. Studying the same patients with both tracers would have been optimal to minimize inter-individual variation in tumour biology, dissemination patterns and length of disease. Second, only a small fraction of patients in our cohort were scanned for disease relapse evaluation, which in theory should be the most promising referral indication, since putative lesions are located in the pelvic area with high background on [^68^Ga]Ga-PSMA-11 PET. However, suspected disease relapse only represents a fraction of PSMA PET referrals at our department. Third, we lack histological confirmation of our findings. However high correlation between imaging and histopathologic findings has been previously demonstrated for PSMA tracers [46, 47]. Finally, although all multiparametric images were based on 30-min D-WB PET data, the dynamic image acquisition protocols were not entirely identical. In a more elegant study setup, we would have preferred identical D-WB PET acquisition protocols.

In conclusion, it is possible to perform D-WB PSMA PET scans that generate lesion and tissue *Ki* values in a clinical setting by using a multiparametric acquisition protocol on standard FOV PET scanners. Both [^68^Ga]Ga-PSMA-11 and [^18^F]PSMA-1007 can be used for lesion detection, with parametric PSMA Ki images showing superior lesion TBR. However, in our small cohort, *Ki* images did not uncover any additional lesions. For quantitative whole-body Ki imaging, [^18^F]PSMA-1007 is the preferred choice due to its predominantly irreversible kinetics in organs and lesions, leading to unbiased quantitative values.

## Supplementary Information

**Additional file 1.**.** Table S1**: VOI delineation methodology. **Figure S1**: Example of target and background delineation.** Figure S2**: Akaike information criterion (AIC) results comparison between the fit for an irreversible and reversible 2-compartment model for both tracers.** Figure S3A**: Kinetic analysis for the liver and spleen for both tracers.** Figure S3B**: Kinetic analysis for the healthy bone and muscle for both tracers.* Figure S3C*: Kinetic analysis for the lesions in the lymph nodes and in the bone for both tracers.** Figure S4**: Correlation between multiparametric Ki values and manual TAC-based Ki estimates using PMOD’s PKIN module.** Figure S5**: Two examples of “false-positive” findings. Both [68Ga]Ga-PSMA-11 scans.** Figure S6**: A case of HCC identified in the patient cohort, D-WB scan with [68Ga]Ga-PSMA-11. Figure S7: Relations of prostate and bladder activity for both tracers.** Figure S8**: Distribution of analysed volumes of interest in benign ganglia with active PSMA uptake.** Figure S9**: Distribution of SUVmax values in the analysed volumes of interest in both healthy organs; and for malignant lesions in the prostate, lymph nodes and skeletal structures.

## Data Availability

Within the restrictions applied by the EU GDPR, all data are available from the authors upon reasonable request.

## References

[1] Hassanipour-Azgomi S, Mohammadian-Hafshejani A, Ghoncheh M, Towhidi F, Jamehshorani S, Salehiniya H (2016). Incidence and mortality of prostate cancer and their relationship with the Human Development Index worldwide. Prostate Int..

[2] Schröder FH, Hugosson J, Roobol MJ, Tammela TL, Ciatto S, Nelen V (2012). Prostate-cancer mortality at 11 years of follow-up. New Engl J Med.

[3] Ceci F, Oprea-Lager DE, Emmett L, Adam JA, Bomanji J, Czernin J (2021). E-PSMA: the EANM standardized reporting guidelines v1.0 for PSMA-PET. Eur J Nucl Med Mol Imaging..

[4] Farolfi A, Calderoni L, Mattana F, Mei R, Telo S, Fanti S (2021). Current and emerging clinical applications of PSMA PET diagnostic imaging for prostate cancer. J Nucl Med.

[5] Kinoshita Y, Kuratsukuri K, Landas S, Imaida K, Rovito PM, Wang CY (2006). Expression of prostate-specific membrane antigen in normal and malignant human tissues. World J Surg..

[6] Sheikhbahaei S, Werner RA, Solnes LB, Pienta KJ, Pomper MG, Gorin MA (2019). Prostate-specific membrane antigen (PSMA)-targeted PET imaging of prostate cancer: an update on important pitfalls. Semin Nucl Med..

[7] Wester HJ, Schottelius M (2019). PSMA-targeted radiopharmaceuticals for imaging and therapy. Semin Nucl Med..

[8] Haupt F, Dijkstra L, Alberts I, Sachpekidis C, Fech V, Boxler S (2020). 68 Ga-PSMA-11 PET/CT in patients with recurrent prostate cancer-a modified protocol compared with the common protocol. Eur J Nucl Med Mol Imaging..

[9] Alberts I, Sachpekidis C, Gourni E, Boxler S, Gross T, Thalmann G (2020). Dynamic patterns of [68 Ga]Ga-PSMA-11 uptake in recurrent prostate cancer lesions. Eur J Nucl Med Mol Imaging..

[10] Sachpekidis C, Kopka K, Eder M, Hadaschik BA, Freitag MT, Pan L (2016). 68Ga-PSMA-11 dynamic PET/CT imaging in primary prostate cancer. Clin Nucl Med..

[11] Sachpekidis C, Eder M, Kopka K, Mier W, Hadaschik BA, Haberkorn U (2016). (68)Ga-PSMA-11 dynamic PET/CT imaging in biochemical relapse of prostate cancer. Eur J Nucl Med Mol Imaging..

[12] Sachpekidis C, Pan L, Hadaschik BA, Kopka K, Haberkorn U, Dimitrakopoulou-Strauss A (2018). 68 Ga-PSMA-11 PET/CT in prostate cancer local recurrence: impact of early images and parametric analysis. Am J Nucl Med Mol Imaging.

[13] Ringheim A, Campos Neto GC, Anazodo U, Cui L, da Cunha ML, Vitor T (2020). Kinetic modeling of 68 Ga-PSMA-11 and validation of simplified methods for quantification in primary prostate cancer patients. EJNMMI Res..

[14] Sachpekidis C, Afshar-Oromieh A, Kopka K, Strauss DS, Pan L, Haberkorn U (2020). 18 F-PSMA-1007 multiparametric, dynamic PET/CT in biochemical relapse and progression of prostate cancer. Eur J Nucl Med Mol Imaging..

[15] Strauss DS, Sachpekidis C, Kopka K, Pan L, Haberkorn U, Dimitrakopoulou-Strauss A (2021). Pharmacokinetic studies of [68 Ga]Ga-PSMA-11 in patients with biochemical recurrence of prostate cancer: detection, differences in temporal distribution and kinetic modelling by tissue type. Eur J Nucl Med Mol Imaging..

[16] Wen J, Zhu Y, Li L, Liu J, Chen Y, Chen R (2021). Determination of optimal 68 Ga-PSMA PET/CT imaging time in prostate cancers by total-body dynamic PET/CT. Eur J Nucl Med Mol Imaging..

[17] Schmuck S, Mamach M, Wilke F, von Klot CA, Henkenberens C, Thackeray JT (2017). Multiple time-point 68Ga-PSMA I&T PET/CT for characterization of primary prostate cancer: value of early dynamic and delayed imaging. Clin Nucl Med..

[18] Karakatsanis NA, Lodge MA, Tahari AK, Zhou Y, Wahl RL, Rahmim A (2013). Dynamic whole-body PET parametric imaging: I. Concept, acquisition protocol optimization and clinical application. Phys. Med Biol.

[19] Karakatsanis NA, Lodge MA, Zhou Y, Wahl RL, Rahmim A (2013). Dynamic whole-body PET parametric imaging: II. Task-oriented statistical estimation. Phys Med Biol..

[20] Patlak CS, Blasberg RG, Fenstermacher JD (1983). Graphical evaluation of blood-to-brain transfer constants from multiple-time uptake data. J Cereb Blood Flow Metab..

[21] Patlak CS, Blasberg RG (1985). Graphical evaluation of blood-to-brain transfer constants from multiple-time uptake data. Generalizations. J Cereb Blood Flow Metab.

[22] Rahmim A, Lodge MA, Karakatsanis NA, Panin VY, Zhou Y, McMillan A (2019). Dynamic whole-body PET imaging: principles, potentials and applications. Eur J Nucl Med Mol Imaging..

[23] Dimitrakopoulou-Strauss A, Pan L, Sachpekidis C (2022). Parametric imaging with dynamic PET for oncological applications: protocols, interpretation, current applications and limitations for clinical use. Semin Nucl Med.

[24] Dimitrakopoulou-Strauss A, Pan L, Sachpekidis C (2021). Kinetic modeling and parametric imaging with dynamic PET for oncological applications: general considerations, current clinical applications, and future perspectives. Eur J Nucl Med Mol Imaging..

[25] Dias AH, Pedersen MF, Danielsen H, Munk OL, Gormsen LC (2021). Clinical feasibility and impact of fully automated multiparametric PET imaging using direct Patlak reconstruction: evaluation of 103 dynamic whole-body 18 F-FDG PET/CT scans. Eur J Nucl Med Mol Imaging..

[26] Fahrni G, Karakatsanis NA, Di Domenicantonio G, Garibotto V, Zaidi H (2019). Does whole-body Patlak (18)F-FDG PET imaging improve lesion detectability in clinical oncology?. Eur Radiol..

[27] Dias AH, Hansen AK, Munk OL, Gormsen LC (2022). Normal values for 18 F-FDG uptake in organs and tissues measured by dynamic whole body multiparametric FDG PET in 126 patients. EJNMMI Res..

[28] Tao Y, Peng Z, Krishnan A, Zhou XS (2011). Robust learning-based parsing and annotation of medical radiographs. IEEE Trans Med Imaging..

[29] Dias AH, Pigg D, Smith AM, Shah V, Gormsen LC, Munk OL. Clinical validation of a population-based input function for dynamic whole-body 18F-FDG multiparametric PET imaging using a standard injector. In: 34th Annual Congress of the European Association of Nuclear Medicine: Eur J Nucl Med Mol Imaging; 2021. p. (Suppl 1) 198–9.

[30] Mittlmeier LM, Brendel M, Beyer L, Albert NL, Todica A, Zacherl MJ (2021). Feasibility of different tumor delineation approaches for 18 F-PSMA-1007 PET/CT imaging in prostate cancer patients. Front Oncol.

[31] Reader AJ, Verhaeghe J (2014). 4D image reconstruction for emission tomography. Phys Med Biol..

[32] Yao S, Feng T, Zhao Y, Wu R, Wang R, Wu S (2021). Simplified protocol for whole-body Patlak parametric imaging with 18 F-FDG PET/CT: feasibility and error analysis. Med Phys.

[33] Chalampalakis Z, Stute S, Filipović M, Sureau F, Comtat C (2021). Use of dynamic reconstruction for parametric Patlak imaging in dynamic whole body PET. Phys Med Biol..

[34] Logan J, Fowler JS, Volkow ND, Wolf AP, Dewey SL, Schlyer DJ (1990). Graphical analysis of reversible radioligand binding from time-activity measurements applied to [N-11C-methyl]-(-)-cocaine PET studies in human subjects. J Cereb Blood Flow Metab.

[35] Akaike H (1974). A new look at the statistical model identification. IEEE Trans Autom Control..

[36] Eder M, Schäfer M, Bauder-Wüst U, Hull WE, Wängler C, Mier W (2012). 68Ga-complex lipophilicity and the targeting property of a urea-based PSMA inhibitor for PET imaging. Bioconjugate Chem.

[37] Cardinale J, Schäfer M, Benešová M, Bauder-Wüst U, Leotta K, Eder M (2017). Preclinical evaluation of 18 F-PSMA-1007, a new prostate-specific membrane antigen ligand for prostate cancer imaging. J Nucl Med.

[38] Fendler WP, Eiber M, Beheshti M, Bomanji J, Ceci F, Cho S (2017). 68 Ga-PSMA PET/CT: joint EANM and SNMMI procedure guideline for prostate cancer imaging: version 1.0. Eur J Nucl Med Mol Imaging..

[39] Derlin T, Weiberg D, von Klot C, Wester HJ, Henkenberens C, Ross TL (2016). 68 Ga-PSMA I&T PET/CT for assessment of prostate cancer: evaluation of image quality after forced diuresis and delayed imaging. Eur Radiol..

[40] Uprimny C, Kroiss AS, Decristoforo C, Fritz J, Warwitz B, Scarpa L (2017). Early dynamic imaging in 68 Ga- PSMA-11 PET/CT allows discrimination of urinary bladder activity and prostate cancer lesions. Eur J Nucl Med Mol Imaging..

[41] Arnfield EG, Thomas PA, Roberts MJ, Pelecanos AM, Ramsay SC, Lin CY (2021). Clinical insignificance of [18 F]PSMA-1007 avid non-specific bone lesions: a retrospective evaluation. Eur J Nucl Med Mol Imaging..

[42] Grünig H, Maurer A, Thali Y, Kovacs Z, Strobel K, Burger IA (2021). Focal unspecific bone uptake on [18 F]-PSMA-1007 PET: a multicenter retrospective evaluation of the distribution, frequency, and quantitative parameters of a potential pitfall in prostate cancer imaging. Eur J Nucl Med Mol Imaging..

[43] Hoberück S, Löck S, Borkowetz A, Sommer U, Winzer R, Zöphel K (2021). Intraindividual comparison of [68 Ga]-Ga-PSMA-11 and [18 F]-F-PSMA-1007 in prostate cancer patients: a retrospective single-center analysis. EJNMMI Res.

[44] Rauscher I, Krönke M, König M, Gafita A, Maurer T, Horn T (2020). Matched-pair comparison of 68 Ga-PSMA-11 PET/CT and 18 F-PSMA-1007 PET/CT: frequency of pitfalls and detection efficacy in biochemical recurrence after radical prostatectomy. J Nucl Med.

[45] Vollnberg B, Alberts I, Genitsch V, Rominger A, Afshar-Oromieh A (2022). Assessment of malignancy and PSMA expression of uncertain bone foci in [18F]PSMA-1007 PET/CT for prostate cancer-a single-centre experience of PET-guided biopsies. Eur J Nucl Med Mol Imaging..

[46] Hofman MS, Lawrentschuk N, Francis RJ, Tang C, Vela I, Thomas P (2020). Prostate-specific membrane antigen PET-CT in patients with high-risk prostate cancer before curative-intent surgery or radiotherapy (proPSMA): a prospective, randomised, multicentre study. Lancet.

[47] Maurer T, Gschwend JE, Rauscher I, Souvatzoglou M, Haller B, Weirich G (2016). Diagnostic efficacy of (68)Gallium-PSMA positron emission tomography compared to conventional imaging for lymph node staging of 130 consecutive patients with intermediate to high risk prostate cancer. J Urol.

