# Supplementary material for: Multiparametric dynamic whole-body PSMA PET/CT using [68Ga]Ga-PSMA-11 and [18F]PSMA-1007
Source: EJNMMI Res. 2023 Apr 15;13:31. doi: 10.1186/s13550-023-00981-8 (PMC10105814; doi:10.1186/s13550-023-00981-8)
Supplement: Supplementary file 1 — Additional file 1.. Table S1: VOI delineation methodology. Figure S1: Example of target and background delineation. Figure S2: Akaike information criterion (AIC) results comparison between the fit for an irreversible and reversible 2-compartment model for both tracers. Figure S3A: Kinetic analysis for the liver and spleen for both tracers. Figure S3B: Kinetic analysis for the healthy bone and muscle for both tracers. Figure S3C: Kinetic analysis for the lesions in the lymph nodes and in the bone for both tracers. Figure S4: Correlation between multiparametric Ki values and manual TAC-based Ki estimates using PMOD’s PKIN module. Figure S5: Two examples of “false-positive” findings. Both [68Ga]Ga-PSMA-11 scans. Figure S6: A case of HCC identified in the patient cohort, D-WB scan with [68Ga]Ga-PSMA-11. Figure S7: Relations of prostate and bladder activity for both tracers. Figure S8: Distribution of analysed volumes of interest in benign ganglia with active PSMA uptake. Figure S9: Distribution of SUVmax values in the analysed volumes of interest in both healthy organs; and for malignant lesions in the prostate, lymph nodes and skeletal structures. [file 13550_2023_981_MOESM1_ESM.pdf]

Multiparametric dynamic whole-body PSMA PET/CT using [^{68}Ga]Ga-PSMA-11 and [^{18}F]PSMA-1007

André H. Dias[1], Mads R. Jochumsen[1,3], Helle D. Zacho[2,4], Ole L. Munk[1,3]*, Lars C. Gormsen[1,3]*

1 Department of Nuclear Medicine & PET Centre, Aarhus University Hospital, Aarhus, Denmark

2 Department of Nuclear Medicine and Clinical Cancer Research Centre, Aalborg University Hospital, Aalborg, Denmark

3 Department of Clinical Medicine, Aarhus University; Aarhus, Denmark

4 Department of Clinical Medicine, Aalborg University, Aalborg, Denmark

*equal contribution

Corresponding author: André H. Dias (andre.dias@auh.rm.dk)

SUPPLEMENTAL MATERIAL

Supplemental Table 1: VOI delineation methodology

**For all healthy areas care was taken to ensure that the outlined VOIs corresponded to areas not affected by pathology**	
**Liver**	Spherical VOI, 20 mm in radius, placed centrally in an area of healthy hepatic tissue.
**Spleen**	Spherical VOI, 10 mm in radius, placed centrally in an area of healthy splenic tissue.
Parotid gland	Spherical VOI, 10 mm in radius, placed centrally in the right parotid gland. Pmod's isocontour tool (50% Max) was then executed to obtain the final area to analyse.
Lacrimal gland	Spherical VOI, 10 mm in radius, placed around the right lacrimal gland. Pmod's isocontour tool (50% Max) was then executed to obtain the final area to analyse.
**Bone**	Cubic VOI, 10 mm sides, placed centrally in the body of a healthy lumbar vertebra (predominantly L5). In cases of disseminate malignant disease of the vertebral body, if no healthy vertebra could be easily segmented an area of healthy bone was delineated in the left upper arm. For the kinetic analysis a thoracic vertebra was delineated using the same method.
**Muscle**	Spherical VOI, 10 mm in radius, placed in an area of paravertebral muscle in the thorax.
**Bladder**	Spherical VOI, 20 mm in radius, placed centrally in the bladder
**Ganglia**	Spherical VOI, placed around the target. Pmod's isocontour tool (50% Max) was then executed to obtain the final area to analyse.
**Lesions**	Spherical VOI, placed around the target lesion. Pmod's isocontour tool (55% Max) was then executed to obtain the final area to analyse.
**Backgrounds**	An elongated ROI, delineated on the axial plane, in the vicinity of the target lesions, in at least 3 consecutive slices.

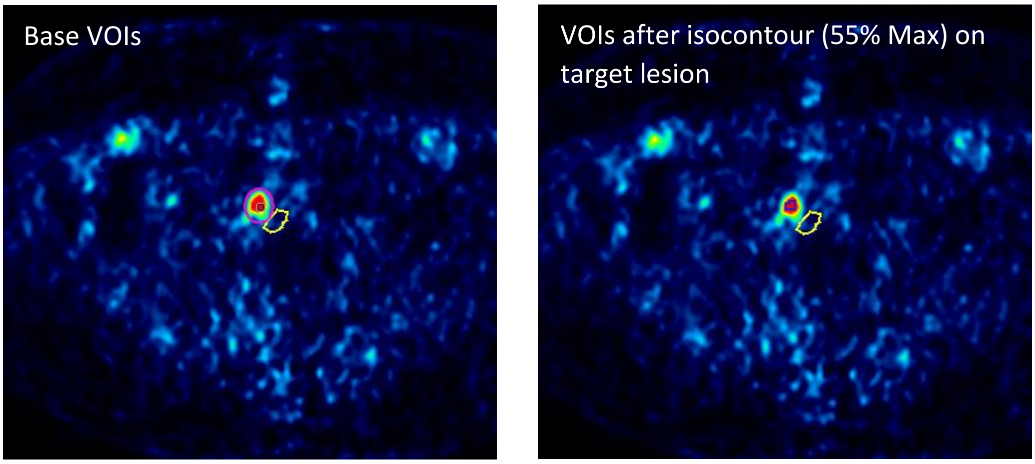

Supplemental Figure 1: Example of target (pink and blue) and background (yellow) delineation, in this case of a metastatic lymph node.

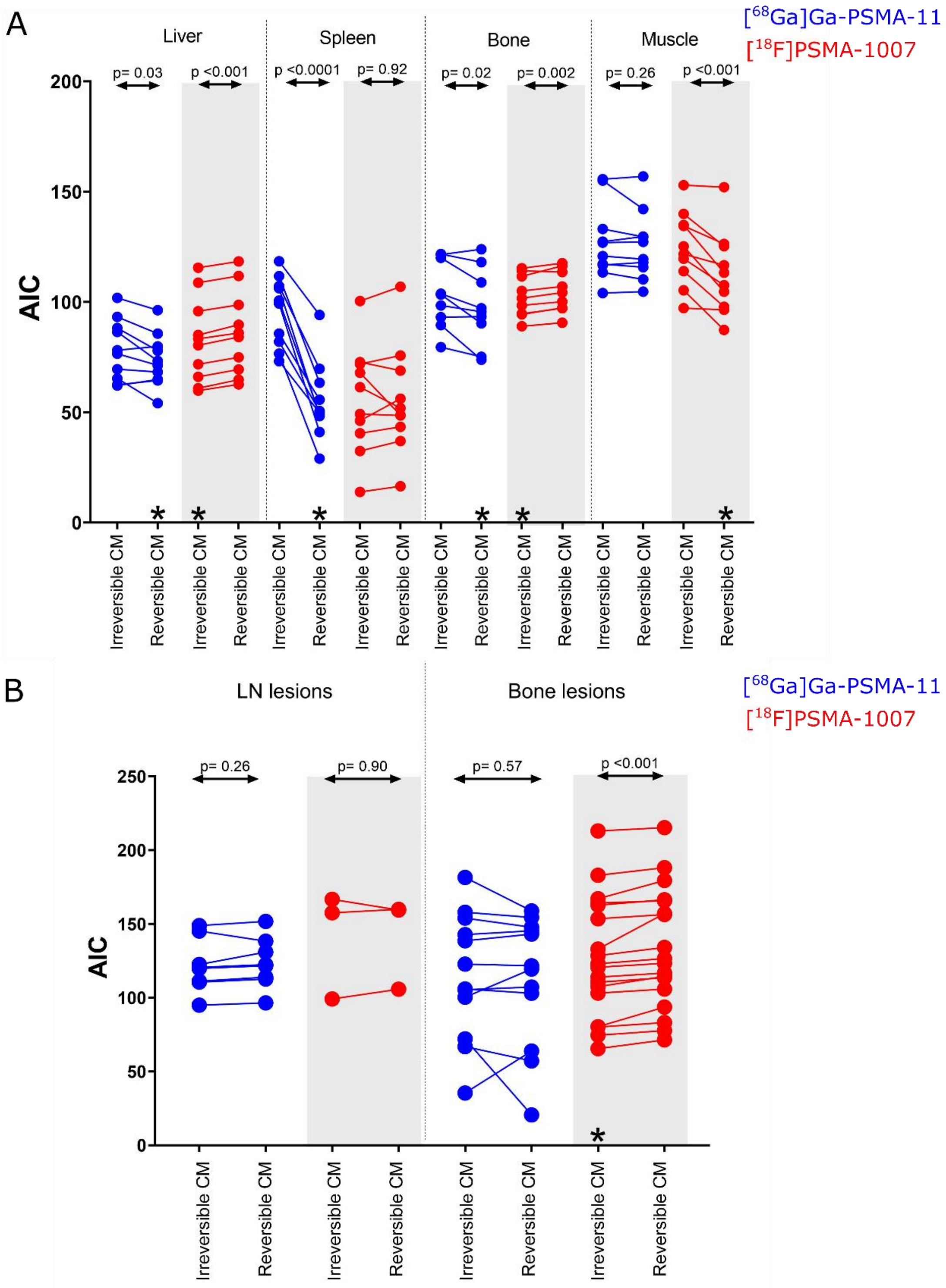

Supplemental Figure 2: Akaike information criterion (AIC) results comparison between the fit for an irreversible and reversible 2-compartment model (CM) for both tracers ([68Ga]Ga-PSMA-11 in blue, [18F]PSMA-1007 in red). Above the two-headed arrows is noted the result of p-value for the statistical T-Test between the irreversible and the reversible model. * Denotes which CM of the pair had a superior performance (lower AIC) when p <0.05.

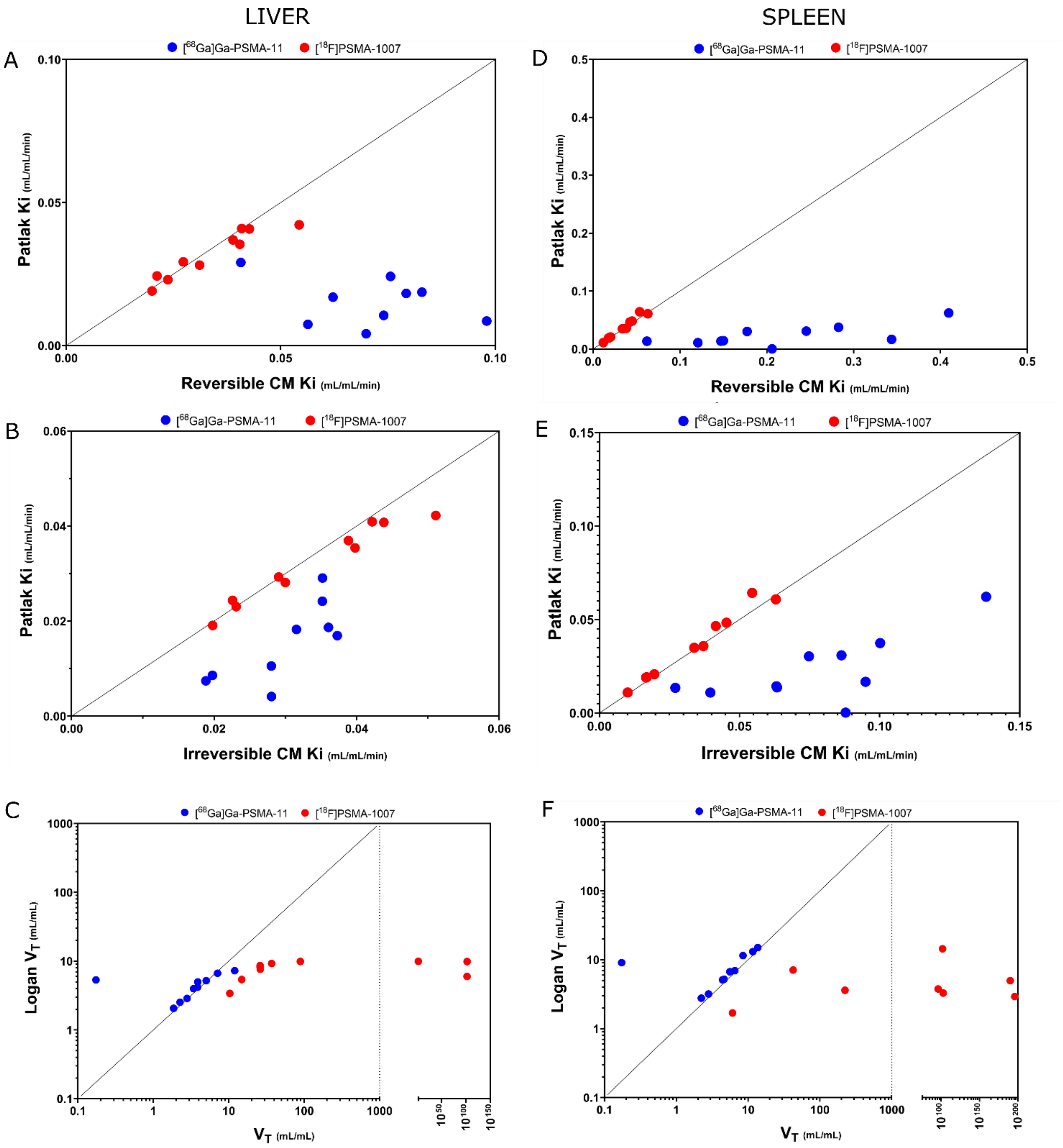

Supplemental Figure 3A: Kinetic analysis for the liver (left column) and spleen (right column) for both tracers ([^{68}Ga]Ga-PSMA-11 in blue, [^{18}F]PSMA-1007 in red). Diagonal lines represent the line of identity. A and D) Represents the relation between the Patlak Ki calculated from a reversible 2-compartment model (2CM) with Ki from the Patlak model. B and E) Represents the relation between the Ki calculated from an irreversible 2CM (k_4=0) with the Ki from the Patlak model. C and F) Represents the relation between the total distribution volume (V_T) calculated from a reversible 2CM with V_T from the Logan model.

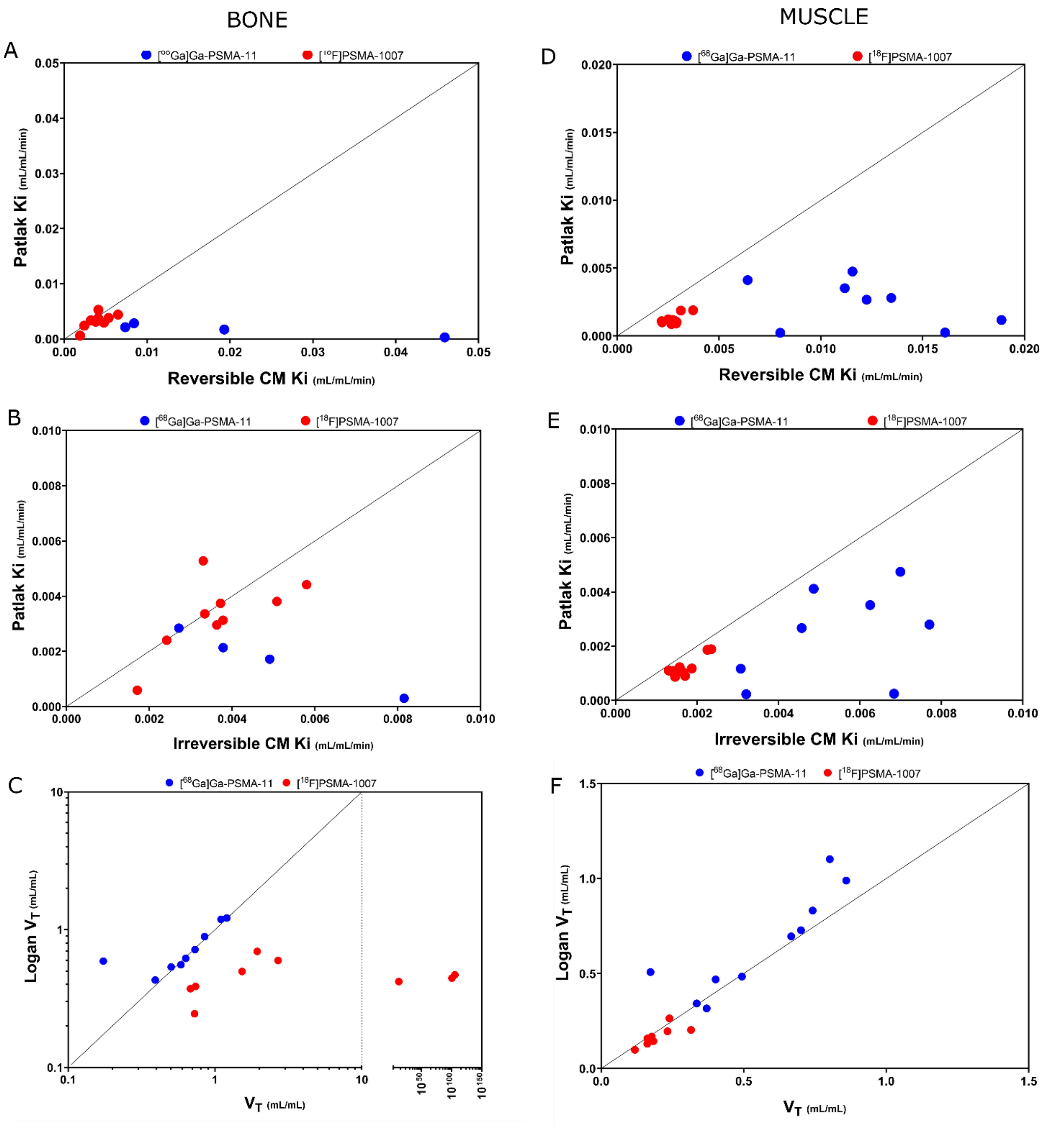

Supplemental Figure 3B: Kinetic analysis for the healthy bone (left column) and muscle (right column) for both tracers ([^{68}Ga]Ga-PSMA-11 in blue, [^{18}F]PSMA-1007 in red). Diagonal lines represent the line of identity. A and D) Represents the relation between the Patlak Ki calculated from a reversible 2-compartment model (2CM) with Ki from the Patlak model. B and E) Represents the relation between the Ki calculated from an irreversible 2CM (k_4=0) with the Ki from the Patlak model. C and F) Represents the relation between the total distribution volume (V_T) calculated from a reversible 2CM with V_T from the Logan model.

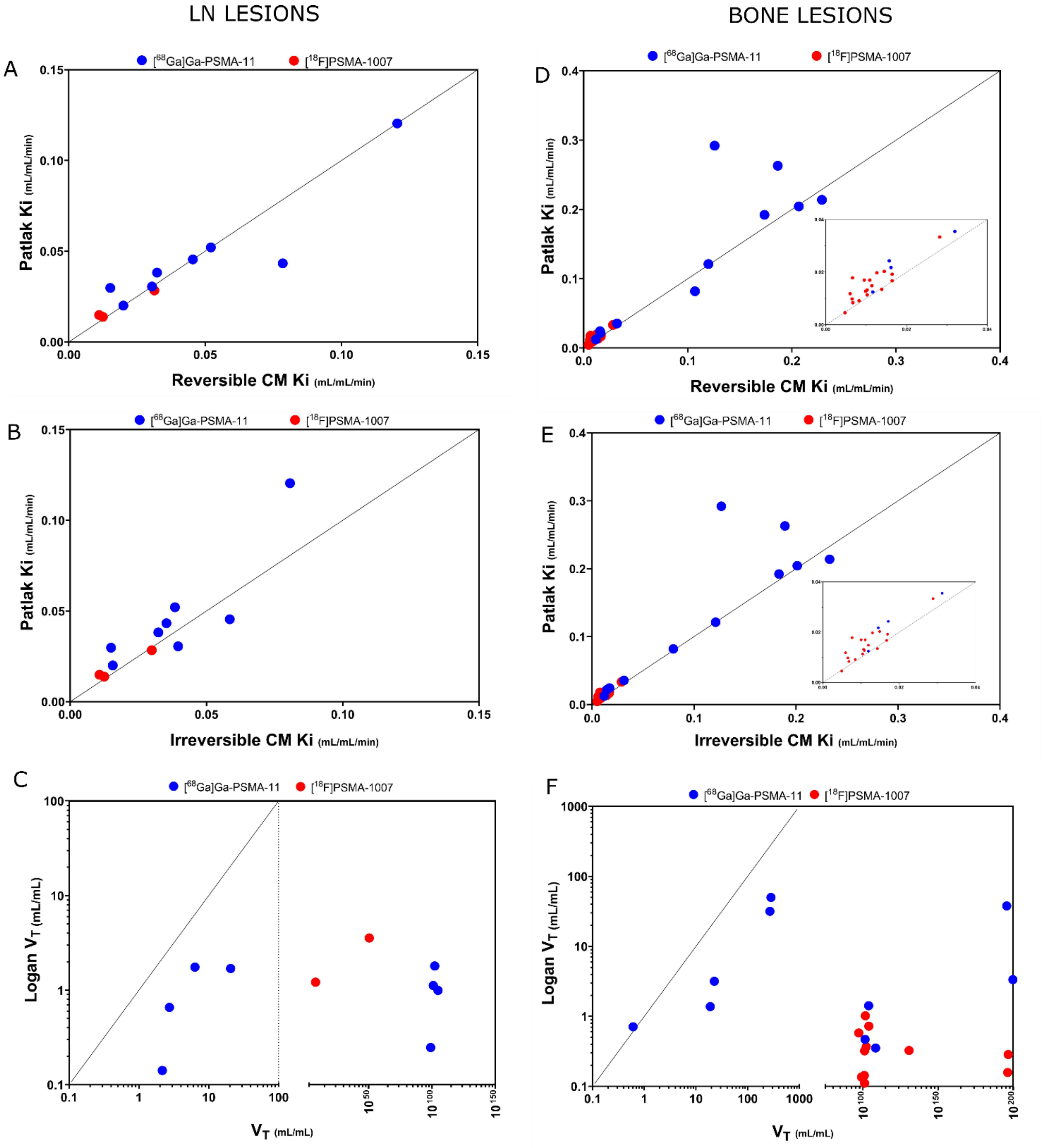

Supplemental Figure 3C: Kinetic analysis for the lesions in the lymph nodes (left column) and in the bone (right column) for both tracers ([68Ga]Ga-PSMA-11 in blue, [18F]PSMA-1007 in red). Diagonal lines represent the line of identity. A and D) Represents the relation between the Patlak Ki calculated from a reversible 2-compartment model (2CM) with Ki from the Patlak model. B and E) Represents the relation between the Ki calculated from an irreversible 2CM (k_4=0) with the Ki from the Patlak model. C and F) Represents the relation between the total distribution volume (V_T) calculated from a reversible 2CM with V_T from the Logan model.

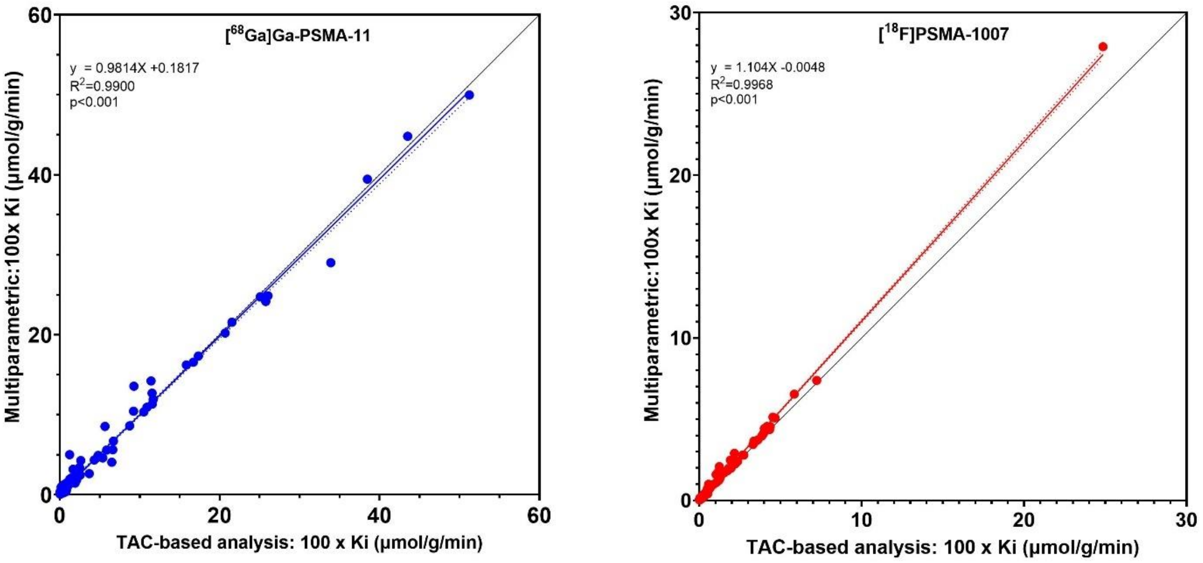

Supplemental Figure 4: Correlation between multiparametric Ki values and manual image-based Ki estimates using PMOD's PKIN module. On the left in blue the correlation for [^{68}Ga]Ga-PSMA-11 (Pearson r^2 =0.99), on the right in red the correlation for [^{18}F]PSMA-1007 (Pearson r^2 =0.99). Manual TAC-based estimates which resulted in fits with negative parametric values were removed to adhere to the non-negativity constraints used in the automatic direct multiparametric calculation.

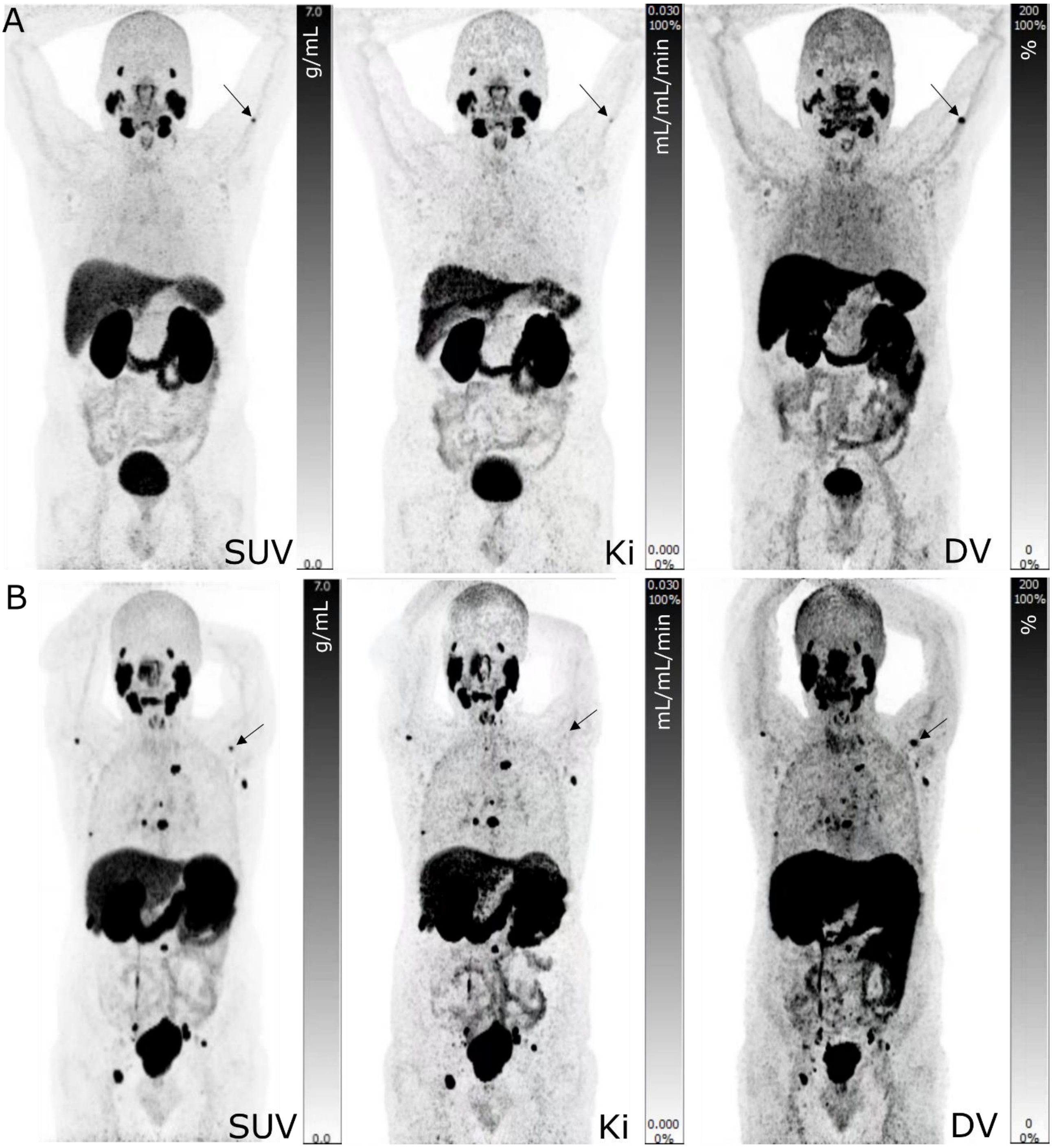

Supplemental Figure 5: Two examples of “false-positive” findings. Both [^{68}Ga]Ga-PSMA-11 scans. Note the SUV avid foci showing very low uptake on Ki images, and conversly intense uptake on DV (marked by arrows). While in patient A it is possible to state on SUV images that this would be a unlikely localization for a single metastasis from prostate cancer, in patient B, on MIP images, the lesion can easily be misdiagnosed as an extra bone metastasis. However the absence of activity on the Ki images demonstrates that this particular lesion is a “false positive” finding.

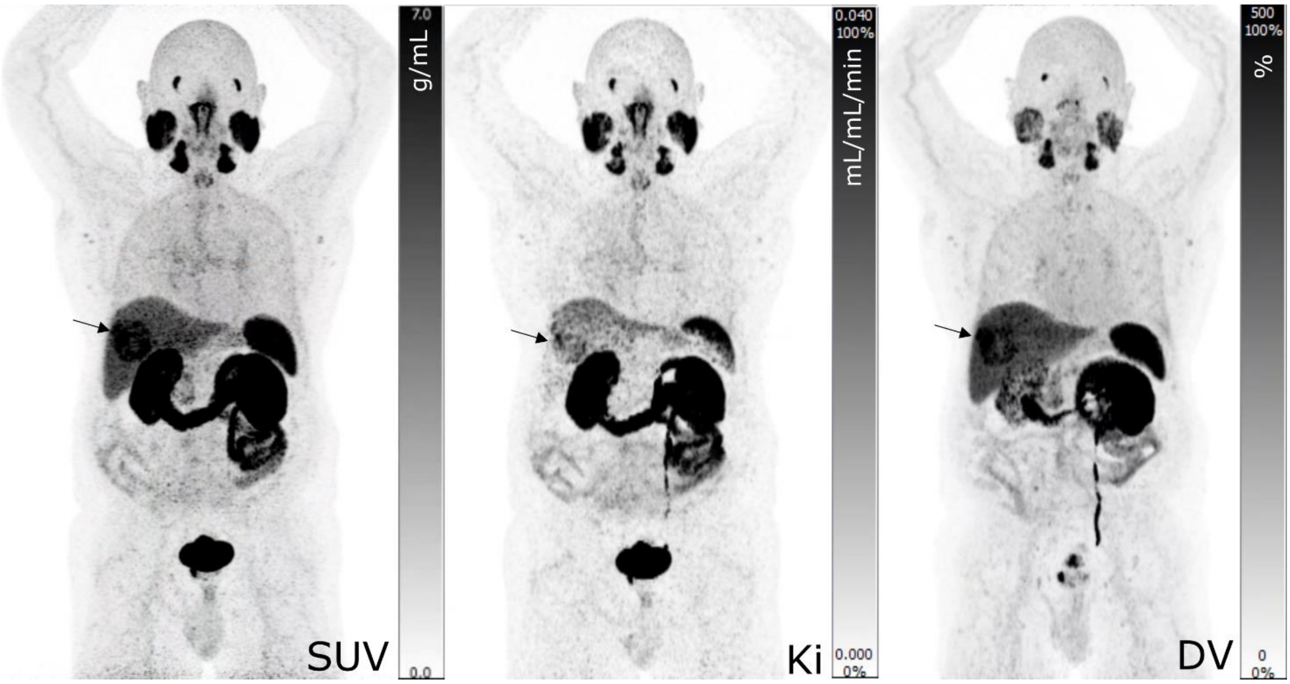

Supplemental Figure 6: A 78 year old patient with newly diagnosed prostata cancer, Gleason 3+4, PSA 11, clinical T1c, was referred to PSMA PET/CT for primary staging. Diagnostic CT showed a suspicious finding in the liver. [^{68}Ga]Ga-PSMA-11 PET/CT scan showed increased uptake on the right side of the prostate, compatible with the known malignacy. Furthermore increased PSMA uptake was seen in the large tumour in the right liver lobe (arrows), suspicious of primary liver malignancy (hepatocelular carcinoma). The intensity scales of the parametric reconstructions were adjusted in order to optimize visualization of the liver malignancy. In the parametric reconstructions, the hepatocellular carcinoma appears to be more easily identified on DV images. This is probably due to the reversible nature of the PSMA uptake in this type of tumor as PSMA uptake in HCC is most likely related to the tumor neovasculature [1,2].

1. Chang SS, O'Keefe DS, Bacich DJ, Reuter VE, Heston WD, Gaudin PB. Prostate-specific membrane antigen is produced in tumor-associated neovasculature. Clinical Cancer Research. 1999;5.
2. Kesler M, Levine C, Hershkovitz D, Mishani E, Menachem Y, Lerman H, et al. 68 Ga-PSMA is a novel PET-CT tracer for imaging of hepatocellular carcinoma: A prospective pilot study. Journal of Nuclear Medicine 2019;60. doi:10.2967/jnumed.118.214833.

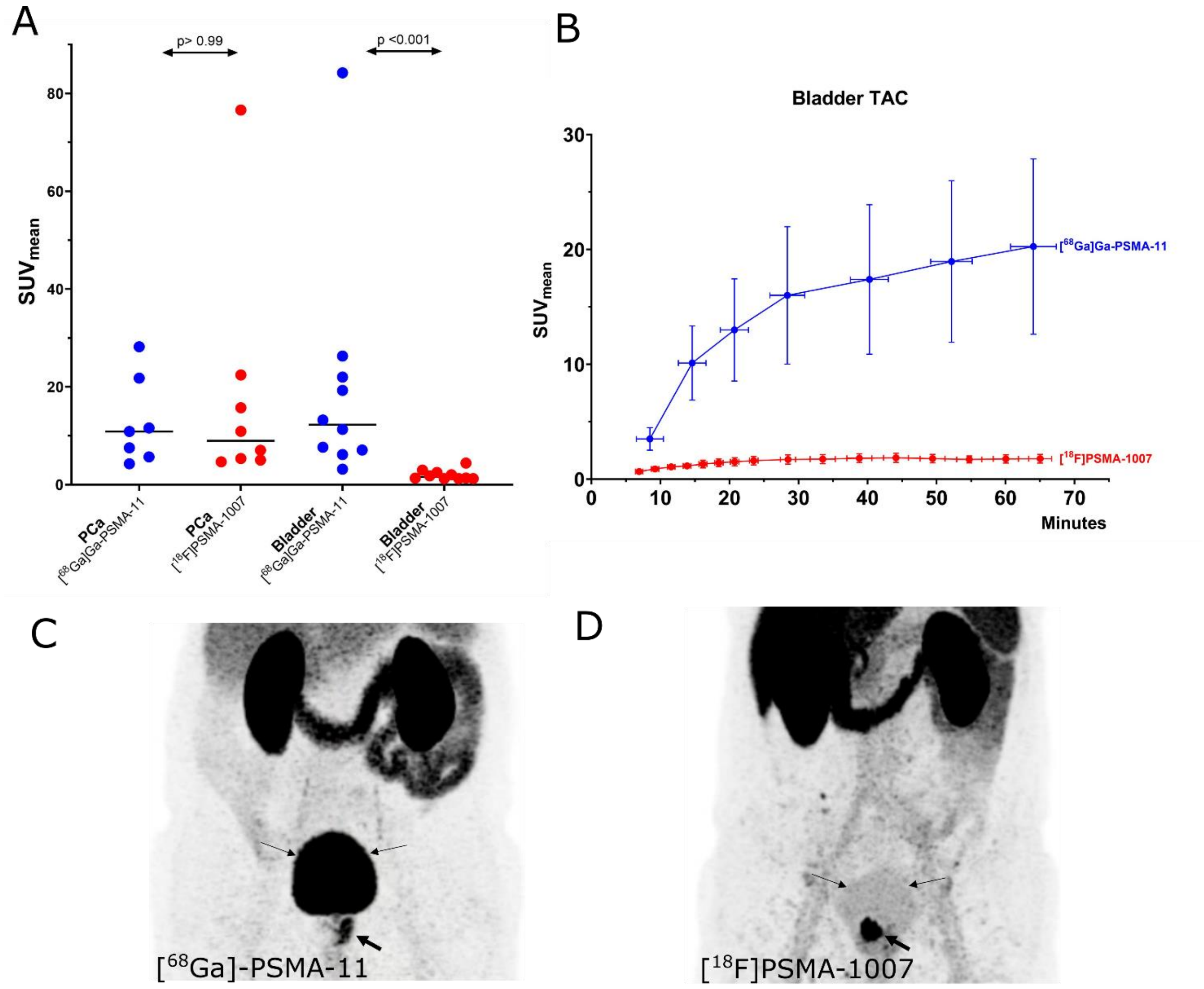

Supplemental Figure 7: A) Plot of the SUV_{mean} activity measured at the primary prostate tumour (PCa) and the bladder. Note the significant difference in bladder activity observed between tracers. [^{68}Ga]Ga-PSMA-11 in blue, [^{18}F]PSMA-1007 in red. B) Time-activity-curves representing of SUV_{mean} in the bladder for both tracers. C) An example of the SUV image of the pelvic area of one of the patients scanned with [^{68}Ga]Ga-PSMA-11. Note intense activity in the bladder. D) An example of the SUV image of the pelvis of a patient scanned with [^{18}F]PSMA-1007 showing much lower tracer activity in the bladder. C) and D) Thick arrows denote the PCa, thin arrows denote the bladder.

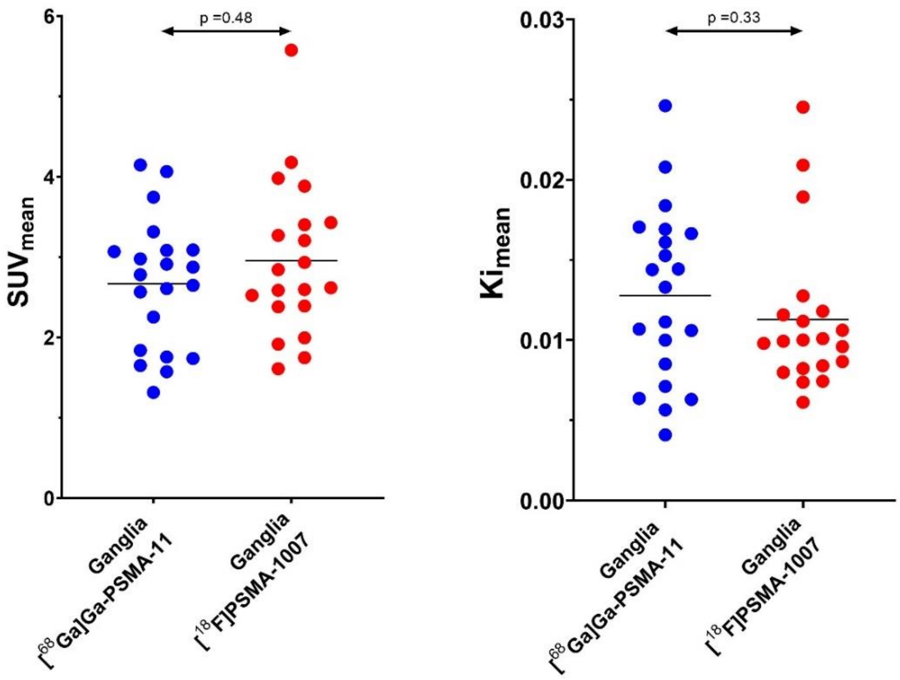

Supplemental Figure 8: Distribution of analysed volumes of interest in benign ganglia with active PSMA uptake; Represented are SUV_{mean} (on the left) and Ki_{mean} (on the right) for both tracers. [^{68}Ga]Ga-PSMA-11 in represented in blue, [^{18}F]PSMA-1007 in red.

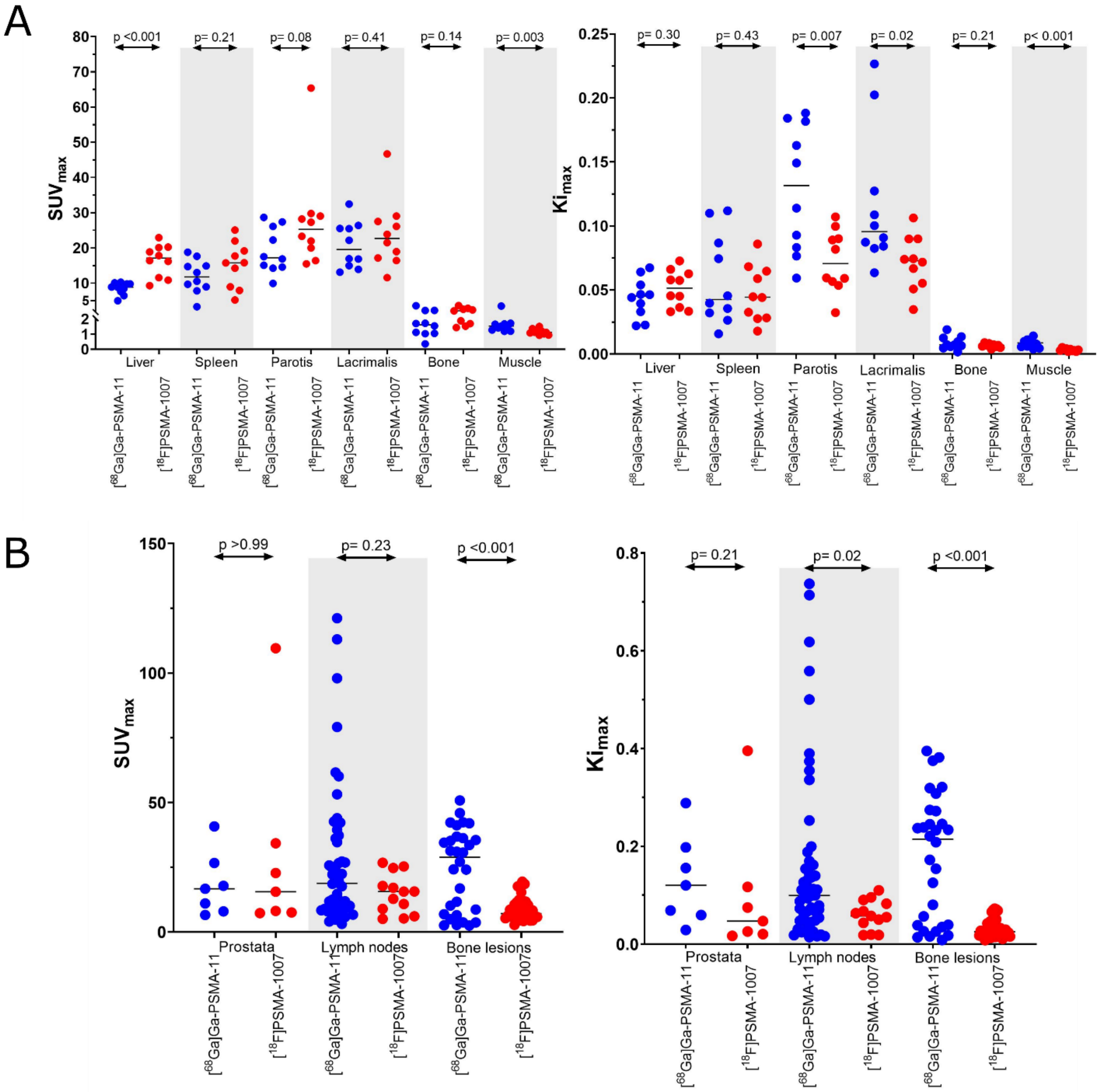

Supplemental Figure 9: Distribution of analysed volumes of interest (A) in healthy organs; (B) for malignant lesions in the prostata, lymph nodes and skeletal structures. Represented are SUV_{max} (on the left) and Ki_{max} (on the right) for both tracers. [^{68}Ga]Ga-PSMA-11 in represented in blue, [^{18}F]PSMA-1007 in red.
